# Effects of Pyrogallol on Growth and Cytotoxicity of Wild-Type and *katG* Mutant Strains of *Vibrio vulnificus*

**DOI:** 10.1371/journal.pone.0167699

**Published:** 2016-12-09

**Authors:** Ju Young Lim, Choon-Mee Kim, Joon Haeng Rhee, Young Ran Kim

**Affiliations:** 1 College of Pharmacy and Research Institute of Drug Development, Chonnam National University, Chonnam National University, Gwangju, Republic of Korea; 2 Premedical Sciences, Chosun University Medical School, Gwangju, Republic of Korea; 3 Clinical Vaccine Research and Development Center, Department of Microbiology, Chonnam National University Medical School, Gwangju, Republic of Korea; Beijing Institute of Microbiology and Epidemiology, CHINA

## Abstract

*Vibrio vulnificus* is a causative agent of fatal septicemia and necrotic wound infection and the pathogen infection became an important public health problem in many counties. *Vibrio vulnificus* causes RtxA1 toxin-induced acute cell death. We tried to identify natural products that inhibit the acute cytotoxicity of *V*. *vulnificus* using a lactate hydrogenase assay. A polyphenol pyrogallol protected HeLa cells from *V*. *vulnificus*-induced cytotoxicity. Pyrogallol also decreased the growth of *V*. *vulnificus*; this inhibitory effect was more significant during log phase than stationary phase. To further elucidate the inhibitory mechanism, pyrogallol-induced toxicity was compared between a *V*. *vulnificus* catalase-peroxidase mutant (*katG*^*−*^) and the isogenic wild-type MO6-24/O strains. No growth was observed for the *katG*^*−*^ mutant in the presence of pyrogallol (50 μg/mL) even after 24 h, whereas the wild-type strain demonstrated growth recovery following a prolonged lag phase. Pyrogallol-mediated growth inhibition of the *katG*^*−*^ mutant strain was partially rescued by exogenous catalase treatment. These results indicate that the mechanism by which pyrogallol inhibits the growth and cytotoxicity of *V*. *vulnificus* likely involves polyphenol-induced prooxidant damage. Taken together, these results suggest that pyrogallol has potential for development as a new paradigm drug to treat infectious diseases.

## Introduction

*Vibrio vulnificus* is a halophilic, estuarine bacterium that causes fatal septicemia and necrotic wound infections [1, 2]. *V*. *vulnificus* infection exhibits a broad pathogenic spectrum, a fulminating course, and a high mortality rate (> 50%) with death occurring within days [3]. Thus, this pathogen represents a good model organism for the study of bacterial septicemia. Global warming appears to be contributing to the current worldwide increase in the frequency and geographical extent of *Vibrio* infections [4]. Previously, we have reported that the major virulence factor of *V*. *vulnificus* is repeats-in-toxin A1 (RtxA1), which induces the programmed necrotic death of host cells [5–7]. To identify compounds with protective activity against *V*. *vulnificus* infection, we conducted a screen of various natural products [3, 8] and found that resveratrol reduces *V*. *vulnificus* pathogenesis by interfering with its adhesion to host cells and inhibiting the production of RtxA1 [3]. Polyphenols have also been reported to have antibacterial activity against many bacteria including *Vibrio* species [9]. The health-promoting effects of polyphenols are generally attributed to their antioxidant action [10]. A polyphenol pyrogallol (benzene-1,2,3-triol) has been shown to possess both anti- and pro-oxidant properties [11]. The prooxidant activity of pyrogallol (i.e., the ability to generate reactive oxygen species such as hydrogen peroxide) may be an important beneficial mechanism as an anti-infection drug [11]. Accordingly, the pharmacological activities of pyrogallol have been studied in infectious diseases; in particular, pyrogallol has been suggested to be a potential inhibitor of *Helicobacter pylori* urease and *Vibrio harveyi* quorum sensing [12]. In the present study, we investigated the effects of pyrogallol on the growth and cytotoxicity of *V*. *vulnificus*. In addition, we evaluated pyrogallol-induced toxicity in wild-type (wt) *V*. *vulnificus* and in a catalase-peroxidase (*katG*^*−*^) mutant strain.

## Materials and Methods

### Bacterial strains and reagents

*V*. *vulnificus* strains were grown in heart infusion (HI) broth (Difco, Becton-Dickinson, Bedford, MA, USA) in a shaking incubator at 200 rpm and 37°C. *V*. *vulnificus* MO6-24/O, a clinical isolate was provided by J. Glenn Morris, Jr., of the University of Maryland [13]. MO6-24/O has been used as a standard cytotoxic strain in *V*. *vulnificus* pathogenesis study and the complete genome sequence was annotated [14]. A catalase-peroxidase deletion mutant (*katG*^*−*^) was constructed in *V*. *vulnificus* wild type (wt) strain MO6-24/O using a counter-selection strategy and the suicide vector pKAS32 [15, 16]. Polymerase chain reaction (PCR) was used to confirm the internal deletion (172–1,793 base pair) within the *katG* gene composed of 2,172 nucleotides. Pyrogallol, catalase, and hydrogen peroxide (H_2_O_2_) (Sigma-Aldrich, St. Louis, MO, USA) were dissolved in phosphate-buffered saline (PBS).

### Cell culturing

HeLa cells (Korea Cell Line Bank, Seoul, Korea) were cultured in Dulbecco's Modified Eagle's Medium (DMEM; Welgene, Kyeongsan-si, Korea) supplemented with 10% heat-inactivated fetal bovine serum (Gibco, Carlsbad, CA, USA) at 37°C in an incubator with a 5% CO_2_ atmosphere.

### Trypan blue staining of HeLa cells infected with *V*. *vulnificus*

HeLa cells were seeded in a 48-well cell culture plate (5×10^4^ cells/well) and cultured overnight. The cells were treated with serum-free DMEM plus pyrogallol (50 μg/mL) for 1 h prior to *V*. *vulnificus* infection at a multiplicity of infection (MOI) of 20 for 2 h. The HeLa cells were stained with a Trypan blue solution (Sigma-Aldrich) for 10 min, followed by washing with PBS. The Cell images were acquired using an inverted microscope with a digital camera (Nikon, Tokyo, Japan).

### Measuring the effects of pyrogallol and H_2_O_2_ on *V*. *vulnificus*-induced HeLa cell cytotoxicity

*V*. *vulnificus* cytotoxicity in HeLa cells was measured using a CytoTox96 non-radioactive cytotoxicity assay kit (Promega, Madison, WI, USA) as previously described [3, 5]. HeLa cells were seeded in a 48-well cell culture plate (5 × 10^4^ cells/well) and cultured overnight. The cells were then washed with serum-free DMEM and treated with pyrogallol or H_2_O_2_ for 1 h prior to *V*. *vulnificus* infection at an MOI of 20 for 2h. Lactate dehydrogenase (LDH) released into the supernatant was assayed as a cytotoxicity marker in accordance with the manufacturer’s protocol.

### Determining the effects of pyrogallol and H_2_O_2_ on HeLa cell viability

HeLa cells cultured in 96-well microplates overnight were washed with serum-free DMEM and treated with pyrogallol (20–100 μg/mL) in a 37°C incubator with 5% CO_2_ for 24 h. Cell viability was measured using a 3-(4,5-dimethylthiazol-2-yl)-5-(3-carboxymethoxyphenyl)-2-(4-sulfophenyl)-2H-tetrazolium (MTS) assay (Promega, Madison, WI, USA) in accordance with the manufacturer’s protocol.

HeLa cells were treated with H_2_O_2_ (0.25–1.0 mM) in a 37°C incubator with 5% CO_2_ for 2 h and cytotoxicity was measured using CytoTox96 as described above.

### Measuring the effects of pyrogallol, catalase, and H_2_O_2_ on the growth of *V*. *vulnificus*

*V*. *vulnificus* wild type or the *katG*^−^ mutant strains were grown in a shaking incubator at 37°C overnight and the culture suspensions were diluted 1:1000 into fresh HI broth. The diluted bacterial suspensions were inoculated into 96-well microplates with pyrogallol (20–100 μg/mL) or catalase (10 μg/mL) and then incubated at 37°C for 6 h. Bacterial growth was determined by measuring the absorbance at 600 nm using a SpectraMax 190 microplate reader (Molecular Devices, Sunnyvale, CA, USA). To assay bacterial growth in a shaking incubator for 24 h, the diluted suspensions of *V*. *vulnificus* were cultured in 50-mL tubes and the optical density was measured every 3 h using a Biophotometer (Eppendorf, Hamburg, Germany).

To test the effect of H_2_O_2_ (0.25–1.0 mM) on *V*. *vulnificus* viability, approximately 1 × 10^7^ colony forming units/mL (CFU/mL) of *V*. *vulnificus* wt or the *katG*^−^ mutant strains were cultured in DMEM for 2 h. For enumeration of live bacterial cells, the *V*. *vulnificus* culture suspensions were 10-fold serially diluted with PBS. The serial dilutions (10 μL) were loaded on HI agar plates and incubated in a 37°C incubator overnight.

### Production of a polyclonal antibody against the KatG protein and Western blot analysis of *V*. *vulnificus* KatG and RtxA1

A DNA fragment encoding the *V*. *vulnificus katG* gene was amplified by PCR using a DNA polymerase (TaKaRa, Tokyo, Japan) and cloned into the pGEX-4T expression vector (Amersham Pharmacia Biotech, Inc., Piscataway, NJ, USA). The constructed plasmid was transformed into *E*. *coli* DH5α and the glutathione *S*-transferase (GST)-fusion protein was induced according to the manufacturer’s protocol (Amersham Pharmacia Biotech). The resulting GST-KatG fusion protein was purified by affinity chromatography in accordance with the manufacturer's recommendations (Amersham Pharmacia Biotech). A polyclonal antibody against the GST-KatG fusion protein was produced using New Zealand white rabbits according to previously described methods [17].

*V*. *vulnificus* wt and the *katG*^*−*^ mutant strains were cultured in HI broth with or without pyrogallol (50 μg/mL) or catalase (10 μg/mL) in a 37°C shaking incubator for 6 h or 24 h. For KatG protein analysis, the bacterial pellets (2 × 10^8^ CFU) were suspended in sodium dodecyl sulfate-polyacrylamide gel electrophoresis (SDS-PAGE) sample buffer and boiled in a water bath for 10 min. For RtxA1, 300 μL supernatants from bacteria cultured in HI for 6 h were concentrated using cold acetone. RtxA1 was detected using an anti-rabbit RtxA1 antibody specific to RtxA1-C [7]. Protein detection was conducted using the Western blotting Luminol reagent (Santa Cruz Biotechnology, Dallas, TX, USA) and a C300 chemiluminescence imager (Azure Biosystems, Inc., Dublin, CA, USA), according to a previously described method [7, 17]. Quantitative protein analysis and determination of fold increase in protein levels were performed by densitometric analysis using the ImageJ program (Azure Biosystems).

### Statistical analysis

Statistical differences were evaluated using one way ANOVA. All experiments were repeated three times and the results shown are from representative experiments. All results are presented as the means ± standard error of the mean (SEM).

## Results

### Effect of pyrogallol on *V*. *vulnificus*-induced HeLa cell cytotoxicity

**Fig 1:**
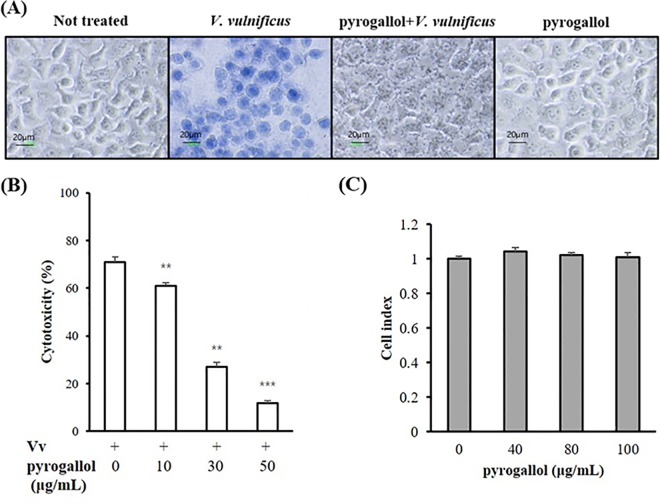
Effect of pyrogallol on *Vibrio vulnificus*-induced cytotoxicity in HeLa cells. (A) HeLa cells pretreated with pyrogallol (50 μg/mL) for 1 h were infected with *V*. *vulnificus* at an MOI of 20 for 2 h. The cells were then stained with Trypan blue and the images were acquired using a microscope with a digital camera. (B) HeLa cells pretreated with pyrogallol (10–50 μg/mL) for 1 h were infected with *V*. *vulnificus* at an MOI of 20 for 2 h. LDH released into the supernatant was assayed as a cytotoxicity marker. (C) HeLa cells were cultured in a 96-well microplate with or without pyrogallol for 24 h and then incubated with MTS at 37°C for 4 h. All values are expressed as the means ± SEM (^**^p < 0.01; ^***^p < 0.001).

Live *V*. *vulnificus* is highly toxic to host cells; this cytotoxicity is caused mainly by the RtxA1 toxin [5]. To identify compounds possessing inhibitory effects on *V*. *vulnificus* cytotoxicity, several natural products were tested using the Trypan blue staining method. Treatment with pyrogallol (50 μg/mL) prevented HeLa cells from becoming damaged by the pathogen (Fig 1A). Pyrogallol did not show any cytotoxicity to host cells (Fig 1A). The inhibitory effect of pyrogallol on *V*. *vulnificus* cytotoxicity was quantified using the LDH assay, which demonstrated that pyrogallol significantly inhibited the cytotoxicity (Fig 1B). The safety of pyrogallol in host cells was also tested using the MTS assay. Pyrogallol did not exhibit any cytotoxicity in HeLa cells (Fig 1C), which indicates that it has relatively low toxicity in human cells.

### Effects of pyrogallol on *V*. *vulnificus* growth in HI broth

**Fig 2:**
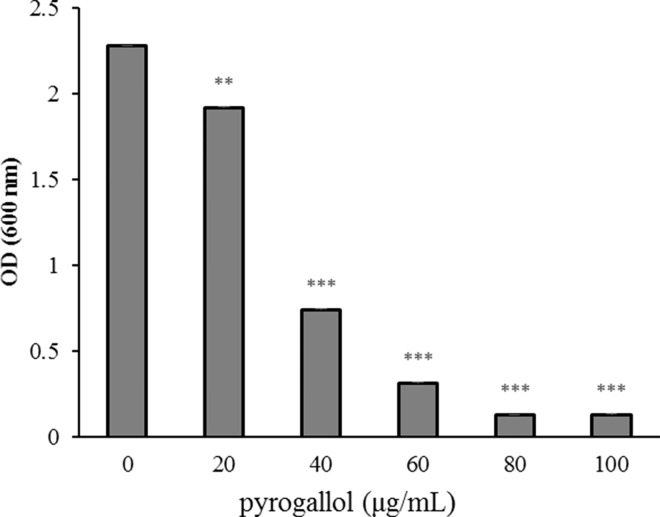
Effects of pyrogallol on *Vibrio vulnificus* growth in HI broth. A *V*. *vulnificus* suspension cultured overnight in HI broth was diluted in 1,000-fold into a 96-well microplate with or without pyrogallol and cultured in a 37°C incubator for 6 h. Bacterial growth was determined by measuring the absorbance at 600 nm using a microplate reader. The data represent the means ± SEM of three experiments (^**^p < 0.01; ^***^p < 0.001).

To investigate the effects of pyrogallol on *V*. *vulnificus* growth, bacterial suspensions cultured overnight were diluted 1,000-fold in HI broth and inoculated into 96-well microplates with or without pyrogallol. The plates were then incubated at 37°C for 6 h and bacterial growth was determined by measuring the absorbance at 600 nm. The results show that pyrogallol (20–100 μg/mL) significantly inhibited *V*. *vulnificus* growth in a dose-dependent manner (Fig 2). The minimum inhibitory concentration (MIC) of pyrogallol on *V*. *vulnificus* growth was approximately 37.6 μg/mL.

### Effects of pyrogallol and catalase on the growth of *V*. *vulnificus* wild type and the *katG*^*−*^ mutant strain

**Fig 3:**
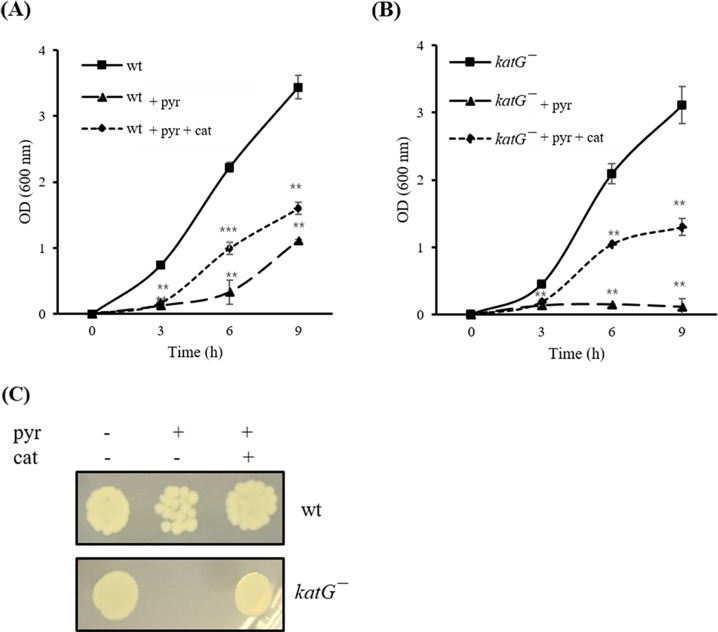
Effect of pyrogallol and catalase on the growth of wild-type (wt) *Vibrio vulnificus* and the *katG*^*−*^ mutant strain. *V*. *vulnificus* wt (A) and the *katG*^−^ mutant (B) cultured overnight in HI broth were diluted 1,000-fold into fresh HI broth with or without pyrogallol (50 μg/mL) or catalase (10 μg/mL). Bacterial growth was determined by measuring the absorbance at 600 nm using a Biophotometer. (C) *V*. *vulnificus* strains were cultured in HI broth with or without pyrogallol or catalase for 24 h and the culture suspensions were then 10-fold serially diluted with PBS. The serial dilutions (10 μL) were then loaded on HI agar plates and incubated at 37°C overnight (pyr: pyrogallol, cat: catalase).

To determine the inhibitory mechanism of pyrogallol, the growth of *V*. *vulnificus* wt and the *katG*^−^ mutant strain was compared in HI broth with or without pyrogallol. The effect of pyrogallol (50 μg/mL) on *V*. *vulnificus* growth was more significant during log phase (3–6 h) than in stationary phase (Fig 3A). In addition, the *katG* mutation itself resulted in a growth defect (Fig 3B). The growth inhibition effect of pyrogallol was more significant in the *katG*^−^ mutant strain than in the wild type strain (Fig 3A and 3B). The *katG*^−^ mutant strain did not exhibit any growth in the presence of pyrogallol (50 μg/mL) after 24 h, whereas the wild type strain showed growth recovery in stationary phase (Fig 3C). This growth inhibition due to pyrogallol was partly reversed by catalase treatment (10 μg/mL) (Fig 3A–3C).

### Effect of H_2_O_2_ on *V*. *vulnificus* and HeLa cells

**Fig 4:**
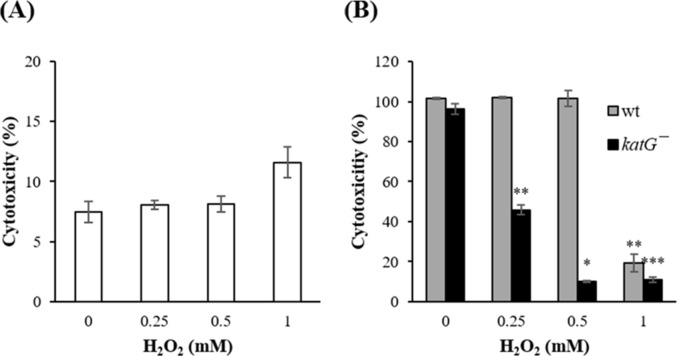
Effect of hydrogen peroxide on *Vibrio vulnificus* growth and HeLa cell cytotoxicity. (A) HeLa cells were treated with H_2_O_2_ (0.25–1 mM) in DMEM for 150 min. (B) HeLa cells pretreated with H_2_O_2_ (0.25–1 mM) for 1 h were infected with *V*. *vulnificus* strains at an MOI of 20 for 150 min. LDH released into the supernatant was assayed as a cytotoxicity marker. All values are expressed as the means ± SEM (^*^p < 0.05; ^**^p < 0.01; ^***^p < 0.001).

The effect of H_2_O_2_ was monitored on the viability of both bacteria and host cells in DMEM. Hydrogen peroxide decreased *V*. *vulnificus* growth (Table 1) but did not show any cytotoxic effect on HeLa cells (Fig 4A). *V*. *vulnificus* cytotoxicity against HeLa cells decreased following H_2_O_2_ treatment (Fig 4B). The effects of H_2_O_2_ were more significant in the *katG*^−^ mutant than in wt *V*. *vulnificus*.

**Table 1:** Effect of hydrogen peroxide (H_2_O_2_) on *V*. *vulnificus* growth.

Strains	H_2_O_2_ (mM)			
	0	0.25	0.5	1.0
**wt (CFU)**	2.8×10^7^±0.46×10^7^	3.8×10^7^±0.31×10^7^*	3.0×10^6^±0.12×10^6^*	**0**
***katG¯*(CFU)**	2.4×10^7^±0.20×10^7^	3.6×10^6^±0.88×10^6^*	6.0×10^6^±0.33×10^6^**	**0**

*V*. *vulnificus* wt and the *katG*^−^ mutant strains were cultured in DMEM with or without H_2_O_2_ for 2 h. The culture suspensions were 10-fold serially diluted. Each dilutions (10 μL) was plated on HI agar plates and incubated at 37°C overnight. Colony numbers were analyzed as viable bacterial numbers. All values are expressed as the means ± SEM (^*^p < 0.05; ^**^p <0.01).

### Effect of pyrogallol and catalase on KatG expression in *V*. *vulnificus*

**Fig 5:**
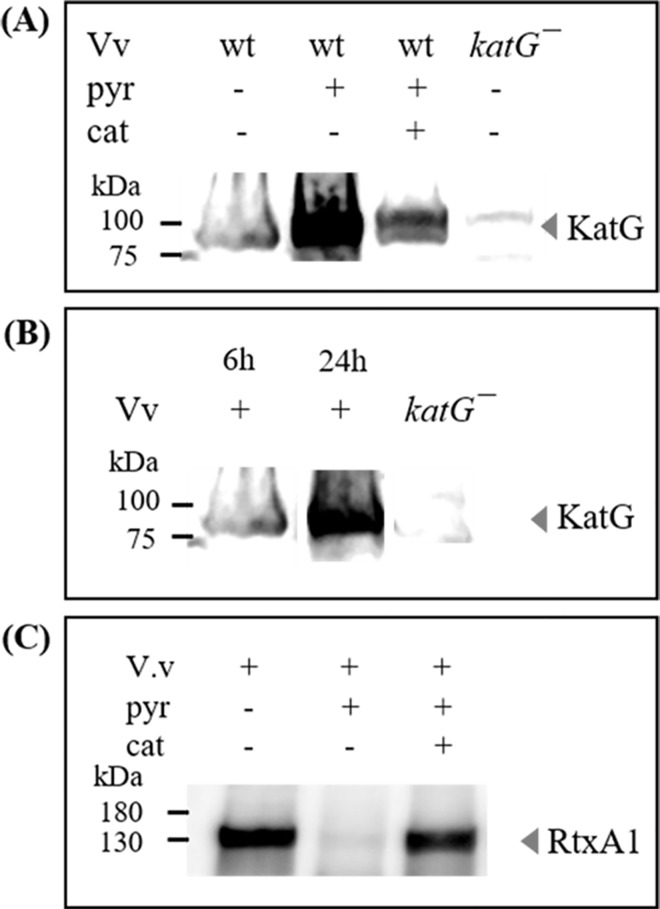
Effect of pyrogallol on the expression of KatG and RtxA1 proteins in *Vibrio vulnificus*. (A) *V*. *vulnificus* wt or *katG*^*−*^ mutant strains were cultured in HI broth with pyrogallol (50 μg/mL) or catalase (10 μg/mL) in a 37°C shaking incubator for 6 h. The bacterial pellets (2 × 10^8^ CFU) were used for KatG protein detection by Western blot analysis. (B) Western blot analysis of KatG in *V*. *vulnificus* bacterial pellets (2 × 10^8^ CFU) cultured for 6 h or 24 h. (C) Western blot analysis of RtxA1 toxin in the supernatants of *V*. *vulnificus* cultured in HI broth for 6 h (pyr: pyrogallol, cat: catalase). (C) *V*. *vulnificus* strains were cultured in HI broth for 6 h. The proteins in culture supernatants (300μl) were precipitated using cold acetone and RtxA1 protein was detected by Western blotting.

To study the role of KatG in the effect of the prooxidant pyrogallol, Western blot analysis was performed using *V*. *vulnificus* cells. At 6 h of incubation, pyrogallol significantly increased the expression of KatG in *V*. *vulnificus* wt (Fig 5A). In contrast, catalase decreased pyrogallol-induced KatG expression (Fig 5A). In addition, KatG expression was higher in a 24 h culture (stationary phase) than in a 6 h culture (log phase) (Fig 5B).

### Effect of pyrogallol and catalase on RtxA1 expression in *V*. *vulnificus*

The RtxA1 toxin, a major cytotoxin of *V*. *vulnificus* was detected in culture supernatants of *V*. *vulnificus* grown in HI broth with pyrogallol or catalase for 6 h. RtxA1 protein was not detected in the HI culture with pyrogallol, which was restored by the addition of catalase (Fig 5C).

## Discussion

*V*. *vulnificus*, a serious opportunistic human pathogen, causes rapidly progressing, fatal septicemia, resulting in a mortality rate of > 50% within a few days of infection. Several recent reports have shown that the incidence of human *Vibrio* illnesses is increasing worldwide; this may be associated with the global warming phenomenon and a rise in sea surface temperatures [18]. In addition, numerous *V*. *vulnificus* isolates have been shown to be resistant to antibiotics routinely prescribed for treating infections caused by this pathogen [19, 20]. Therefore, we have attempted to identify new compounds from natural products with therapeutic activity against *V*. *vulnificus* [3]. The results of the present study show that pyrogallol significantly inhibits the growth of *V*. *vulnificus* and decreases the pathogen-induced cytotoxicity in HeLa cells (Fig 1 and Fig 2). Importantly, pyrogallol did not exhibit any toxicity in the host cells, based on cell morphology and viability (Fig 1A and 1C).

Pyrogallol has been reported to possess both antioxidant and prooxidant properties [11]. In support of this functionality, a mutant strain defective in an oxidative stress-related protein (*katG*^*−*^) did not show any growth in the presence of pyrogallol (Fig 3B). The KatG protein has been reported to be significantly upregulated during the resuscitation of viable but nonculturable *V*. *vulnificus* cells [21]. Here, we confirmed that KatG expression increased in *V*. *vulnificus* during stationary phase (Fig 5B) and in a culture incubated with pyrogallol (Fig 4A). Additionally, the growth inhibition caused by pyrogallol could be partly reversed by co-treatment with exogenous catalase in both *V*. *vulnificus* wt and the *katG*^*−*^ mutant strain (Fig 3). In particular, the prooxidant ability of pyrogallol to generate reactive oxygen species such as H_2_O_2_ has been suggested as an important beneficial mechanism. Our results show that H_2_O_2_ exhibits inhibitory effects on *V*. *vulnificus* growth (Table 1) similar to those mediated by pyrogallol. Catalase partly reduced the effects of pyrogallol (Fig 3). These results indicate that pyrogallol might inhibit *V*. *vulnificus* growth via the production of reactive oxygen species. The growth inhibition in HI broth culture by pyrogallol was more significant in log phase than in stationary phase (Fig 3). Pyrogallol also caused the decrease of RtxA1 expression in HI broth culture for 6 h (Fig 5C), which might result from the growth inhibition.
